# Metabolic Engineering of Oleaginous Yeast *Yarrowia lipolytica* for Overproduction of Fatty Acids

**DOI:** 10.3389/fmicb.2020.01717

**Published:** 2020-07-24

**Authors:** Rishikesh Ghogare, Shulin Chen, Xiaochao Xiong

**Affiliations:** Department of Biological Systems Engineering, Washington State University, Pullman, WA, United States

**Keywords:** *Yarrowia lipolytica*, fatty acid, oleaginous yeast, fatty acyl-CoA, thioesterase, lipid

## Abstract

The oleaginous yeast *Yarrowia lipolytica* has attracted much attention due to its ability to utilize a wide range of substrates to accumulate high lipid content and its flexibility for genetic manipulation. In this study, intracellular lipid metabolism in *Y. lipolytica* was tailored to produce fatty acid, a renewable oleochemical and precursor for production of advanced biofuels. Two main strategies, including blocking activation and peroxisomal uptake of fatty acids and elimination of biosynthesis of lipids, were employed to reduce fatty acid consumption by the native pathways in *Y. lipolytica*. Both genetic modifications improved fatty acid production. However, disruption of the genes responsible for assembly of nonpolar lipid molecules including triacylglycerols (TAGs) and steryl esters resulted in the deleterious effects on the cell growth. The gene *tesA* encoding thioesterase from *Escherichia coli* was expressed in the strain with disrupted *faa* genes encoding fatty acyl-CoA synthetases and *pxa1* encoding peroxisomal acyl-CoA transporter, and the titer of fatty acids resulted in 2.3 g/L in shake flask culture, representing 11-fold improvement compared with the parent strain. Expressing the native genes encoding acetyl-CoA carboxylase (ACC) and hexokinase also increased fatty acid production, although the improvement was not as significant as that with *tesA* expression. Saturated fatty acids including palmitic acid (C16:0) and stearic acid (C18:0) increased remarkably in the fatty acid composition of the recombinant bearing *tesA* compared with the parent strain. The recombinant expressing *tesA* gene resulted in high lipid content, indicating the great fatty acid producing potential of *Y. lipolytica*. The results highlight the achievement of fatty acid overproduction without adverse effect on growth of the strain. Results of this study provided insight into the relationship between fatty acid and lipid metabolism in *Y. lipolytica*, confirming the avenue to reprogram lipid metabolism of this host for overproduction of renewable fatty acids.

## Introduction

Increase in greenhouse gas emissions and volatility in fossil fuel prices have prompted development of alternative chemicals and fuels produced by microorganisms from renewable biomass. Microbial lipids produced from renewable feedstock such as cellulosic sugars can be used as the precursors for sustainable production of fungible fuels including green diesel and biodiesel with high-energy density (Xiong et al., 2012). The model organisms such as *Escherichia coli* and *Saccharomyces cerevisiae* have been intensively engineered for production of lipids and lipid-derived chemicals (Steen et al., 2010). Compared with *E. coli* and *S. cerevisiae* (Ferreira et al., 2018), oleaginous yeasts have superior natural ability to accumulate large amount of lipids, mainly consisting of triacylglycerols (TAGs; Adrio, 2017). Among these oleaginous strains, *Yarrowia lipolytica* is one of the most intensively studied non-conventional yeasts and serves as a model for investigating the mechanism of lipid accumulation (Madzak, 2018). Because of its generally recognized as safe (GRAS) status, *Y. lipolytica* has been metabolically engineered for production of an array of renewable chemicals such as succinic acid (Cui et al., 2017) and triacetic acid lactone (TAL; Markham et al., 2018) and other high-value bioproducts such as β-carotene and limonene (Cao et al., 2016).

In *Y. lipolytica*, TAGs are the main storage forms of fatty acids, and metabolism of fatty acid plays an important role in production and accumulation of lipids (Beopoulos et al., 2012). Fatty acids themselves are valuable oleochemical, and they can be readily converted into fuels such as biodiesel and alkane and other chemicals such as fatty alcohols and long-chain dicarboxylic acids by either chemical or biological means (Steen et al., 2010). In oleaginous strains, TAGs are often stored in the cells as lipid droplets (Gajdoš et al., 2016). However, fatty acids can be secreted out by some strains due to the straight-chain structures of fatty acid molecules (Leber et al., 2015). It is naturally to believe that *Y. lipolytica* can be designed and engineered as a cell factory for efficient production of fatty acids by tailoring intracellular lipid metabolism.

Metabolic engineering strategies have been used to improve production of lipids in *Y. lipolytica*, including overexpressing gene(s) for improvement of precursors supply for fatty acid biosynthesis (“push”), overexpressing genes for fatty acid formation and TAG assembly (“pull”), and deleting genes for β-oxidation (“block”; Liu et al., 2017). Overexpression of two genes, *YlAcc1* and *YlDga1*, encoding the enzymes, acetyl-CoA carboxylase (ACC) and diacylglycerol acyltransferase (DGAT) increased lipid content to 61.7% of dry cell weight (DCW), with lipid yield of 0.195 g/g glucose and productivity of 0.143 g/L/h achieved by *Y. lipolytica* (Tai and Stephanopoulos, 2013). Lipid titers of 39.1 and 55 g/L with maximal lipid productivities of 0.89 and 1 g/L/h have been achieved with evolutionary and rational engineering, respectively (Liu et al., 2015). In *Y. lipolytica*, overexpression of the native gene *YlHxk1* encoding hexokinase enhanced cell growth, reduced filament formation, and improved lipid content when grown on hexose sugars such as glucose and fructose. By engineering NADPH regeneration pathway in *Y. lipolytica*, the strain achieved a productivity of 1.2 g/L/h for lipid accumulation (Qiao et al., 2017). To engineer the strains for production of fatty acids, some of these strategies including improvement of fatty acid biosynthesis and deleting genes for β-oxidation can be adopted because TAG formation is dependent on incorporation of fatty acids as major backbone. However, to develop strains for more efficient production of fatty acids, it is critical to specifically engineer the metabolism of fatty acid based on the understanding of the mechanism underlying formation, activation, utilization, and degradation of fatty acids in the cells.

Considerable efforts have already been made to metabolically engineer the model organism *S. cerevisiae* for overproduction of fatty acids. In the yeast cells such as *S. cerevisiae*, there are cellular compartments to isolate metabolites. Long-chain fatty acids (C16–C18) are mainly degraded through β-oxidation in the organelle, peroxisome. Prior to β-oxidation, fatty acids are activated to fatty acyl-CoA and then transported by ABC transporters into peroxisome (Leber et al., 2015). In *S. cerevisiae*, peroxisomal transporter complex PXA1–2 plays an important role in transporting acyl-CoA into peroxisome (Leber et al., 2015). There are four fatty acyl-CoA synthetases encoded by *faa* genes, namely, *ScFaa1*, *ScFaa2*, *ScFaa3*, and *ScFaa4* in *S. cerevisiae* (Faergeman et al., 2001). In addition, there are two fatty acid transporters ScFat1, which is responsible for uptake and transport of long-chain fatty acids, and ScFat2 with unknown function (Coe et al., 1999). Studies suggest that ScFaa1 and ScFaa4 play a major role in activation of free fatty acids in *S. cerevisiae*. The deletion of *ScFaa1* and *ScFaa4* led to production of 310 mg/L of intracellular and 320 mg/L extracellular fatty acids, and the yield of fatty acids was further improved by blocking β-oxidation (Leber et al., 2015). Moreover, heterologous expression of thioesterase from *Mus musculus* improved fatty acid production and secretion by *S. cerevisiae* (Chen et al., 2014). The elimination of biosynthesis of TAGs and steryl esters in *S. cerevisiae* also increased fatty acid production (Sandager et al., 2002; Valle-Rodríguez et al., 2014). In addition to deletion of *Faa1*, *Faa4*, and *Pox1* gene encoding fatty acyl-CoA oxidase and expression of *tesA* encoding a truncated *E. coli* thioesterase, a hybrid pathway consisting of ATP citrate lyase (ACL) from *M. musculus*, malic enzyme (ME) from *Rhodosporidium toruloides*, and endogenous malate dehydrogenase (Mdh3) with eliminated peroxisomal signal peptide was constructed to enhance generation of acetyl-CoA, and the resultant *S. cerevisiae* strain could produce 10.4 g/L of C16–C18 fatty acids in fed-batch fermentation (Zhou et al., 2016). By combination of metabolic engineering and adaptive laboratory evolution, a Crabtree negative *S. cerevisiae* without ethanol production was developed and further improvement led to production of 33.4 g/L free fatty acids (Dai et al., 2018).

**Figure 1:**
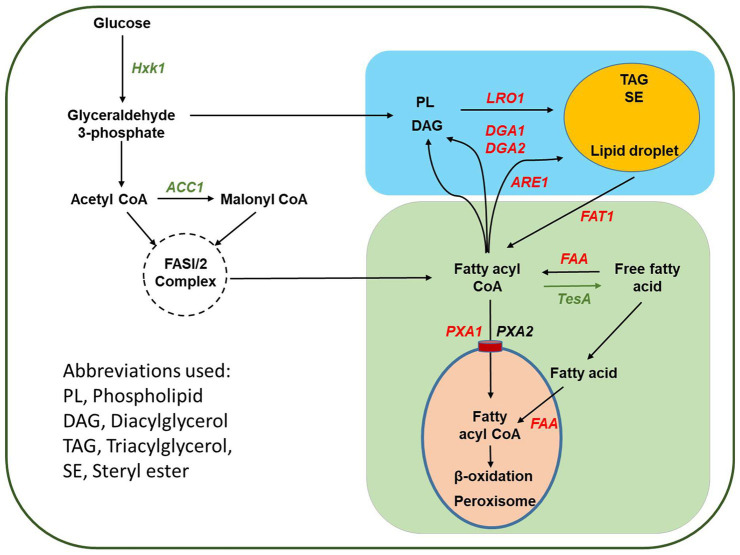
Schematic representation of the pathway design for metabolic engineering of *Yarrowia lipolytica* to produce free fatty acid. The genes to be deleted are shown with red font, whereas the gene targets for overexpression are shown with green font.

In addition to the model organism *S. cerevisiae*, fatty acid metabolism was investigated in *Y. lipolytica*, and the finding of these studies could provide insights to design and engineer efficient cell factories (Figure 1). In *Y. lipolytica*, there were two genes, *YlPxa1* (YALI0A06655g) and *YlPxa2* (YALI0D04246g), with identities to their corresponding genes in *S. cerevisiae*, which were responsible for the transport of fatty acyl-CoA into peroxisome (Dulermo et al., 2015; Leber et al., 2015). Knockout of *YlFaa1* (YALI0D17864g) increased the contents of both total lipids and saturated fatty acids (Wang et al., 2011). In *Y. lipolytica*, *YlFat1* had a different function than *ScFat1*, and it was involved in mobilization of fatty acids from lipid droplets after the hydrolysis of TAGs catalyzed by lipase (Dulermo et al., 2014). However, the other possible *faa* genes encoding fatty acyl-CoA synthetase in *Y. lipolytica* have been neither reported nor targeted for metabolic engineering. As an oleaginous organism, *Y. lipolytica* accumulates high TAG levels. In the final step of assembly, TAGs are either formed by combination of diacylglycerol (DAG) with acyl-CoA, which is catalyzed by DGAT encoded with genes *YlDga1* (YALI0E32769g) and *YlDga2* (YALI0D07986g), or acyl-CoA independent pathway mediated with phospholipid:diacylglycerol acyltransferase (PDAT) encoded by *YlLro1* (YALI0E16797g; Tai and Stephanopoulos, 2013; Figure 1). In lipid droplets, there are also steryl esters. In *Y. lipolytica*, biosynthesis of steryl esters is catalyzed by steryl ester synthase, which is encoded by gene of *YlAre1* (YALI0F06578g), belonging to the acyl-CoA cholesterol acyltransferase family (Koch et al., 2014). Overproduction of fatty acids was achieved by metabolic engineering of *Y. lipolytica* through the combinatorial deletion of one fatty acyl-CoA synthetase gene and elimination of β-oxidation and additional overexpression of intracellular lipases for hydrolysis of TAGs into fatty acids (Ledesma-Amaro et al., 2016).

To develop a highly productive strain for production of fatty acids, it requires optimization of multiple metabolic modules, including increasing acetyl-CoA supply and malonyl-CoA generation, balancing cofactor NADPH, blocking fatty acid degradation, improving fatty acid production, and controlling conversion of fatty acids into lipids in a systematic way (Yan and Pfleger, 2020). This study employed two different strategies, which included blocking activation and peroxisomal uptake of fatty acids and elimination of biosynthesis of TAGs and steryl esters, to limit fatty acid consumption in *Y. lipolytica*. Multiple candidate *faa* genes were identified and disrupted in the host. Due to better performance of cell growth and fatty acid production, the strain with disrupted genes of fatty acyl-CoA synthetases and peroxisomal acyl-CoA transporter was selected for next-step engineering. Fatty acid production was further improved by expressing native genes, *YlAcc1*, *YlHxk1*, and *TesA* gene from *E. coli* in the mutant strain. Expression of *tesA* gene led to great increase in fatty acid production. The results highlight the achievement of fatty acid overproduction without detrimental effects on cell growth, existence of multiple *faa* genes in *Y. lipolytica*, and the importance of biosynthesis of non-polar lipid molecules, although they were previously believed as non-essential pathways in *S. cerevisiae*.

## Materials and Methods

### Strains and Culture Conditions

*E. coli* TOP10 was used as the host strain for cloning of genes and propagation of plasmids (Table 1). *E. coli* carrying plasmids was grown at 37°C on lysogeny broth (LB) medium supplemented with 100 μg/ml ampicillin (Xiong et al., 2016). The yeast strains were derived from *Y. lipolytica* Po4f, a *Ku70*-deleted strain of PO1f (ATCC MYA-2613) that was auxotrophic for both uracil and leucine (Wang et al., 2016). *Y. lipolytica* strains were cultivated in flasks with yeast extract peptone dextrose (YPD) media consisting of 10 g/L of yeast extract (Difco), 20 g/L of peptone (Difco), and 20 g/L of glucose at 28°C on a shaker with a shaking speed of 180 rpm. YPD agar plates were made by adding 15 g/L agar (Difco). Yeast transformants were grown in selective media depending on genotype. The yeast transformants were selected and isolated on agar plates of synthetic media lacking either uracil or leucine. Selective media was composed of 20 g/L glucose and 6.7 g/L yeast nitrogen base YNB without amino acid and with ammonium sulfate (US Biologicals), supplemented with 2.0 g/L of complete supplement of amino acids lacking uracil or leucine (US Biologicals). Agar plates of selective media were prepared by adding 15 g/L agar to broth.

**Table 1:** Strains used in this study.

Strain	Characteristic(s)	Reference or source
*E. coli* Top10	F^−^ *mcr*A Δ(*mrr*-*hsd*RMS-*mcr*BC) φ80*lac*ZΔM15 Δ*lac*X74 *rec*A1 *ara*D139 Δ(*ara leu*) 7697 *gal*U *gal*K *rps*L (StrR) *end*A1 *nup*G	Invitrogen
*E. coli* K12	Wild type, source of *tesA*	*E. coli* Genetic Stock Center (CGSC)
*Y. lipolytica* Po1f	*MatA*, *leu2-270*, *ura3-302*, *xpr2-322*, *axp1-2*	ATCC MYA-2613
*Y. lipolytica* Po4f	*Ku70* knockout, *leu2* ^−^, *ura3* ^−^	(Wang et al., 2016)
*Y. lipolytica* YlR1	*Y. lipolytica* Po4f penta knockout *∆Faa1*, *∆Faa2*, *∆Faa3*, *∆Fat1*, *∆Pxa1*, *leu2* ^−^, *ura3* ^+^	This work
*Y. lipolytica* YlR2	*Y. lipolytica* Po4f quadra knockout *∆Dga1*, *∆Dga2*, *∆Lro1*, *∆Are1*, *leu2* ^−^, *ura3* ^+^	This work
*Y. lipolytica* Po4f Ura	*Y. lipolytica* Po4f, *leu2* ^−^, *ura3* ^+^	This work
*Y. lipolytica* YlRX	*Y. lipolytica* Po4f penta knockout *∆Faa1*, *∆Faa2*, *∆Faa3*, *∆Fat1*, *∆Pxa1*, *leu2* ^−^, *ura3* ^−^	This work
*Y. lipolytica* Po4f Control	*Y. lipolytica* Po4f integrated with linearized plasmid pGR12, *leu2* ^+^, *ura3* ^−^	This work
*Y. lipolytica* YlRX Control	*Y. lipolytica* YlRX integrated with linearized plasmid pGR12, *leu2* ^+^, *ura3* ^−^	This work
*Y. lipolytica* YlRX Acc1	*Y. lipolytica* YlRX overexpressing *Ylacc1*, *leu2* ^+^, *ura3* ^−^	This work
*Y. lipolytica* YlRX Hexo	*Y. lipolytica* YlRX overexpressing *YlHxk1*, *leu2* ^+^, *ura3* ^−^	This work
*Y. lipolytica* YlRX TesA	*Y. lipolytica* YlRX overexpressing *tesA*, *leu2* ^+^, *ura3* ^−^	This work

The media for growing and testing the cells for production of fatty acids contained 80 g/L glucose, 20 g/L yeast extract, 1.36 g/L ammonium sulfate, and 1.7 g/L yeast nitrogen base without amino acid and ammonium sulfate. The cultivation was carried out by inoculating single colonies into culture tubes containing 3 ml media and then incubating at 28°C and 180 rpm for 48 h. The seed cultures were then used to inoculate 50 ml of media in a 250 ml baffled flask, which was cultivated at 28°C and 180 rpm. The initial absorbance at 600 nm (OD_600_) of the culture was adjusted to 0.05.

### DNA Techniques

Plasmid DNA was extracted from *E. coli* by using a GeneJET Plasmid Miniprep Kit from Thermo Fisher Scientific. FastDigest restriction endonucleases were purchased from Thermo Fisher Scientific. To get DNA fragments for cloning, PCR was performed with Q5 high-fidelity DNA polymerase from NEB. Other PCR was routinely carried out by using DreamTaq Green PCR Master Mix from Thermo Scientific. Oligonucleotide primers were synthesized in Invitrogen (Grand Island, NY). The sequence and generated restriction sites of the primers are listed in Supplementary Table S1. The PCR products and plasmids digested by restriction endonucleases were recovered from agarose gel after electrophoresis with GeneJET Gel Extraction kit (Thermo Scientific). The competent cells of *E. coli* were made by CaCl_2_ solution and transformed with DNA by fowling the heat-shock procedure. Prior to transformation of *Y. lipolytica*, the digested DNA fragments were recovered by using DNA Clean & Concentrator kit (Zymo Research, Irvine, CA). *Y. lipolytica* cell was transformed with linearized plasmid or DNA fragment by using Frozen-EZ Yeast Transformation II Kit (Zymo Research, Irvine, CA).

### Construction of Recombinant Strains for Overproducing Fatty Acids

The gene encoding Ku70 in *Y. lipolytica* Po1f was disrupted to enhance the homologous recombinant frequency, and new strain was designated Po4f. Around 1.0-kb DNA fragments of 5' and 3' region of targeted genes to be deleted were amplified with primers (Supplementary Table S1) by PCR and then cloned into the plasmid containing selection marker gene *ura3*. To knockout a set of selected genes in *Y. lipolytica* Po4f, the procedure of the homologous recombination was followed, including *Cre/loxP*-mediated excision of the selection marker *ura3* and curing of the plasmid pJN44-cre carrying the *Cre* gene encoding recombinase.

The 7.27-kb *YlAcc1* gene containing intron was amplified by PCR with primers YlAcc1-F and YlAcc1-R using *Y. lipolytica* genome DNA as template. After digestion of PCR product with SmaI, *YlAcc1* gene was inserted into expression vector pGR12 developed previously (Wang et al., 2016). In the resultant plasmid pGR12 Acc1, expression of the native gene *Acc1* from *Y. lipolytica* was under control of FBA promoter. Similarly, the plasmid pGR52 Hxk was constructed to express native gene *YlHxk1* driven by GPM promoter (Wang et al., 2016). A new vector pJN44 TesA containing TEF promoter with first intron (TEFIN) was developed to express 0.55-kb *tesA* gene encoding leaderless thioesterase I from *E. coli* K12. The vector pGR12 with *leu2* gene as a selection marker was treated by restriction enzyme SpeI. The linearized pGR12 was integrated into *Y. lipolytica* Po4f and YlRX. The restriction enzyme SpeI was used to linearize plasmids pGR12 Acc1 and pGR52 Hxk, whereas plasmid pJN44 TesA was digested with SphI. The DNA fragments containing both *leu2* marker and expression cassettes of cloned genes were integrated into the genome of knockout strain YlRX by transformation with linearized plasmids. Transformants were cultured and isolated on the synthetic agar plates without leucine. The strains used in this study are listed in Table 1.

### Quantification of Lipids and Free Fatty Acids by Gas Chromatography Analysis

Extraction and transesterification of lipids were performed using the method described previously (Xiong et al., 2012). Extracted fatty acid methyl esters (FAMEs) in hexane were used for gas chromatography (GC) analysis with tridecanoic acid (C13:0) as an internal standard. The protocol for extraction of free fatty acids was developed by modifying the lipid extraction method developed by Folch et al. (1957). Cell culture with volume of 0.5 ml was mixed with 0.75 ml chloroform: methanol (2:1) solution. Cells were lysed by using a Mini-BeadBeater with 0.5 mm glass beads (BioSpec Products, Inc., Bartlesville, OK). The mixture was centrifuged at 13,000 rpm for 10 min to separate the organic phase, and then the organic phase was washed with 0.9% NaCl solution. The organic phase containing fatty acids was directly used for analysis with GC. GC analysis was performed using Agilent 7890A GC (Agilent Technologies, Inc., Santa Clara, CA) equipped with a flame ionization detector (FID) and FAMEWAX column (30 m × 320 μm × 0.25 μm; Restek Corporation). One microliter of the sample was injected with a split ratio of 20:1. Injector temperature was set at 250°C, and the initial oven temperature was set at 120°C, ramped up at the rate of 5°C/min to 240°C, and then held for 25 min. The detector temperature was set at 250°C. Fatty acids produced by *Y. lipolytica* were quantified by using the standard curves of individual fatty acids with different contents (Sigma).

### Measurement of Glucose Content, Cell Dry Weight, and Cell Growth

Samples were collected every 24 h for measurement of residual glucose. One milliliter culture was centrifuged at 13,000 rpm. Supernatant was used for determination of residual glucose in the medium. Glucose was analyzed using Dionex ICS-3000 Ion Chromatography equipped with CarboPac TM PA20 analytical column and CarboPac TM PA20 guard column (Xiong et al., 2016). Glucose concentration was quantified by using the external standard method.

The cell growth was determined by measuring the absorbance at 600 nm of the culture using Shimadzu UV-Vis spectrophotometer, UV-2550. For cell dry weight (CDW) measurement, 5 ml of cell culture was collected and centrifuged at 13,000 rpm for 10 min. The pellets were washed twice with 5 ml of distilled water and dried at 104°C, until a consistent weight was obtained (approximately 24 h). The data of weight of dried cell biomass were recorded.

## Results

### Development of Knockout Strains for Producing Fatty Acids

By using the sequences of *faa* genes from *S. cerevisiae* as the reference, four candidate genes, including *YlFaa1* (YALI0D17864g), *YlFaa2* (YALI0E12859g), *YlFaa3* (YALI0F06556g), and *YlFat1* (YALI0E16016g), have been identified for activation of fatty acids into acyl-CoA in *Y. lipolytica* (Leber et al., 2015). All the enzymes encoded by these four genes from *Y. lipolytica* have potential adenosine monophosphate (AMP) binding domains, one of the important characteristics of fatty acyl-CoA synthetases (Shockey et al., 2000), and *YlFaa1* and *Fat1* genes have been identified and studied previously (Wang et al., 2011; Dulermo et al., 2014). The identities of *faa* genes from *Y. lipolytica* with the corresponding genes from *S. cerevisiae* were summarized in Supplementary Table S2. The protein sequence of peroxisomal ABC transporter YlPxa1 (YALI0A06655g) showed around 40% identity with ScPxa1 (Dulermo et al., 2015). These genes were targeted for deletion to impair fatty acid activation and transport of fatty acyl-CoA to peroxisome for β-oxidation in *Y. lipolytica* (Figure 1). Mutant *Y. lipolytica* YlR1 (*∆Faa1*, *∆Faa2*, *∆Faa3*, *∆Fat1*, and *∆Pxa1*) was constructed by combinatorial deletion of the genes encoding fatty acyl-CoA synthetases and a peroxisomal transporter (Table 1). To investigate the relationship between biosynthesis of lipid molecules including TAGs and steryl esters and fatty acid production, a mutant strain *Y. lipolytica* YlR2 (*∆YlDga1*, *∆YlDga2*, *∆YlLro1*, and *∆YlAre1*) bearing multiple disrupted genes involved in lipid assembly was constructed (Table 1).

### Production of Fatty Acids by *Y. lipolytica* Knockouts

**Figure 2:**
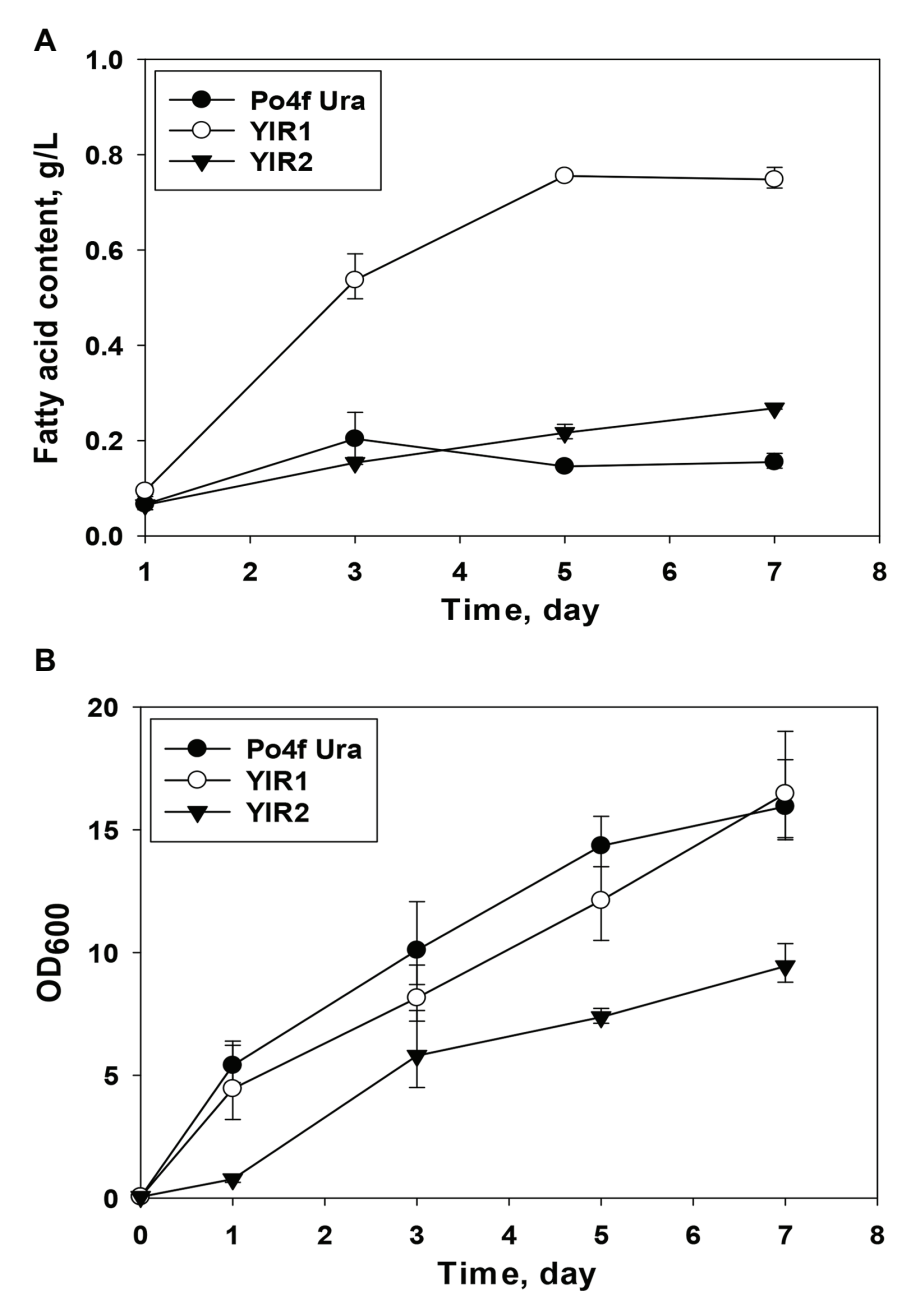
**(A)** Comparison of fatty acid produced by *Y. lipolytica* knockout strains including YlR1 (*∆Faa1*, *∆Faa3*, *∆Faa4*, *∆Fat1*, and *∆Pxa1*) and YlR2 (*∆Dga1*, *∆Dga2*, *∆Lga1*, and *∆Are1*), where Po4f Ura was used as a control strain over the culture period of 7 days. **(B)** Comparison of cell growth of *Y. lipolytica* strains including Po4f Ura, YlR1, and YlR2. The culture was carried out in flasks at 28°C with shaking speed at 180 rpm.

The results of fatty acid content measurement indicated that the mutant *Y. lipolytica* YlR1 could accumulate more fatty acids than both strain YlR2 and the control, Po4f Ura (Figure 2A). The titer of fatty acids produced by *Y. lipolytica* YlR1 reached the highest value (0.755 g/L) on day 5 and kept this similar level for the last 3 days. The fatty acid concentration in *Y. lipolytica* YlR1 was three times over that of YlR2 (Figure 2A). The results show that deletion of genes responsible for fatty acid activation and peroxisomal transport successfully improved fatty acid production by *Y. lipolytica*, but the mutant YlR2 showed no remarkable increase in fatty acid titer. The highest concentration of fatty acids achieved by *Y. lipolytica* YlR2 was at 0.268 g/L, and the titer was only 1.7 folds greater than the control on day 7 (Figure 2A). When the growth of the resultant knockout strains checked, no obvious growth defect was observed in the mutant YlR1 (Figure 2B). However, the growth of *Y. lipolytica* YlR2 was severely affected by blocking pathways for biosynthesis of TAGs and steryl esters (Figure 2B). Both the growth rate and final biomass yield of *Y. lipolytica* YlR2 were much lower than those of the strain YlR1 and control Po4f Ura. At the end of cultivation, OD_600_ of cell culture of *Y. lipolytica* YlR2 was only around half of that of the control strain under the same culture condition (Figure 2B). In contrast, there was no deleterious effect on growth observed in *S. cerevisiae* by the similar genetic manipulations of genes involved in TAG and steryl ester biosynthesis (Sandager et al., 2002; Valle-Rodríguez et al., 2014). Based on the improved cell growth and production performance, strain YIR1 was selected for further improvement of fatty acid production.

### Improvement of Fatty Acid Production by Overexpression of Target Genes

**Figure 3:**
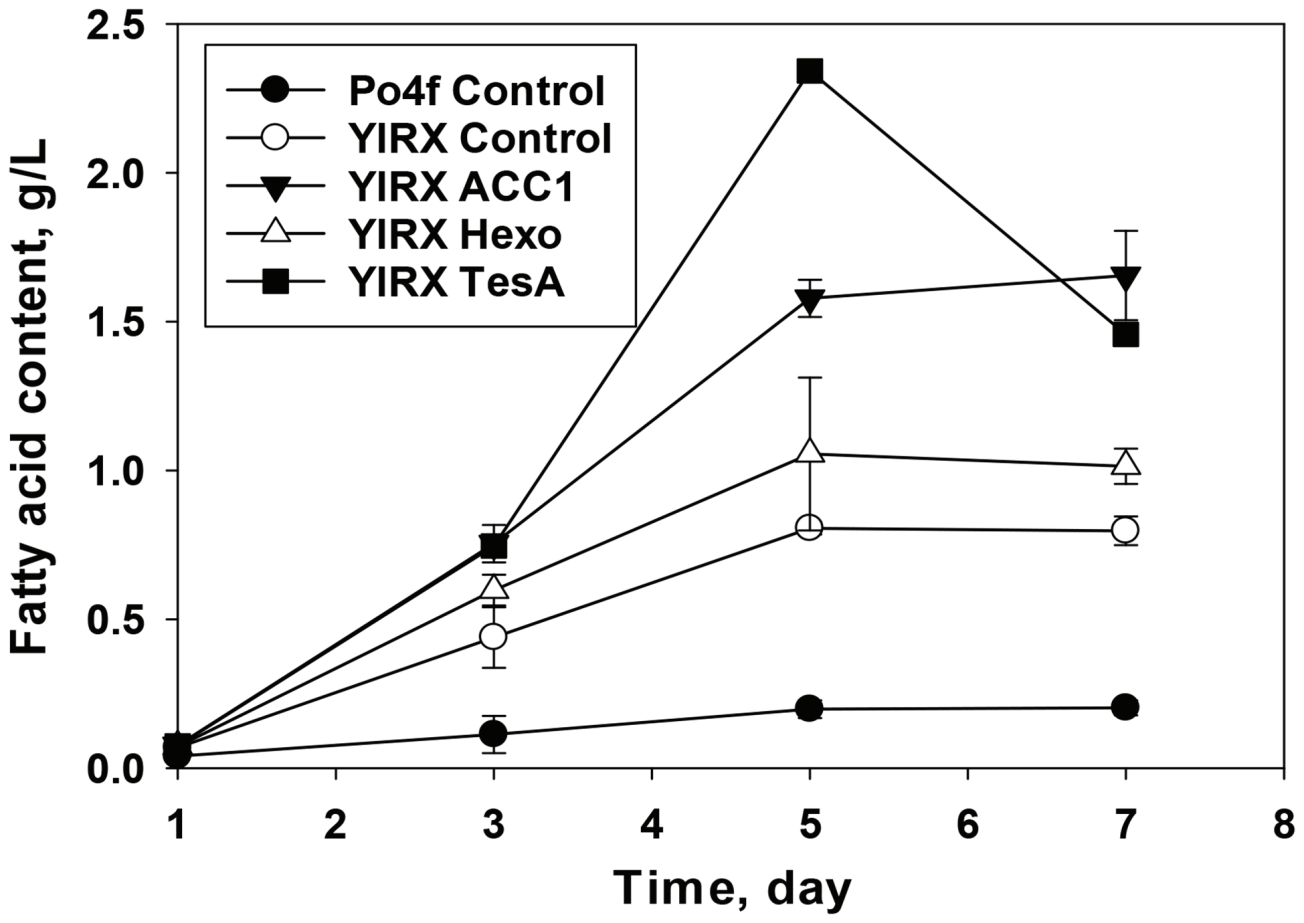
Fatty acid production by *Y. lipolytica* knockout (*∆Faa1*, *∆Faa2*, *∆Faa3*, *∆Fat1*, and *∆Pxa1*) overexpressing *YlAcc1*, *YlHxk1*, and *tesA* from *Escherichia coli*. *Y. lipolytica* YlRX Control and Po4f Control were cultivated under the same conditions. Cultivation was carried out in a shaker at 28°C with shaking speed at 180 rpm.

To further enhance production of fatty acids, three genes, including *YlAcc1*, *YlHxk1*, and *tesA*, from *E. coli* were expressed individually in *Y. lipolytica* mutant (*∆Faa1*, *∆Faa2*, *∆Faa3*, *∆Fat1*, and *∆Pxa1*). The strain expressing a truncated version of *TesA* without signal peptide from *E. coli* produced 2.3 g/L fatty acids on day 5, whereas the control strain Po4f only produced 0.198 g/L fatty acids under the same culture conditions (Figure 3). The concentration of fatty acids in the strain expressing *tesA* declined to 1.45 g/L on day 7. The decrease in fatty acid content after day 5 in strain *Y. lipolytica* YlRX TesA might be due to free fatty acid degradation by other unidentified fatty acyl-CoA synthetases and further utilization for biosynthesis of TAGs and steryl esters when glucose was depleted.

The recombinant *Y. lipolytica* YlRX Acc1 produced fatty acids at the level of 1.57 and 1.65 g/L on day 5 and 7, respectively. The strain YlRX Hexo accumulated ~1 g/L free fatty acids on day 5 and 7. These results showed that expression of *TesA* from *E. coli* in *Y. lipolytica* could improve fatty acid production more significantly than expression of *YlAcc1* and *YlHxk1*. The *YlHxk1* expressed strain showed less improvement in fatty acid accumulation, and this was consistent with the previous report where expression of *YlHxk1* mainly improved lipid production when fructose, instead of glucose, was used as carbon source (Lazar et al., 2014). Extracellular fatty acids secreted by the engineered strains were negligible. There was no obvious growth defect was observed, suggesting high tolerance of *Y. lipolytica* to long-chain (C16–C18) fatty acids.

### Glucose Consumption, Cell Growth, Lipid Production, and Fatty Acid Profiles of Recombinants

**Figure 4:**
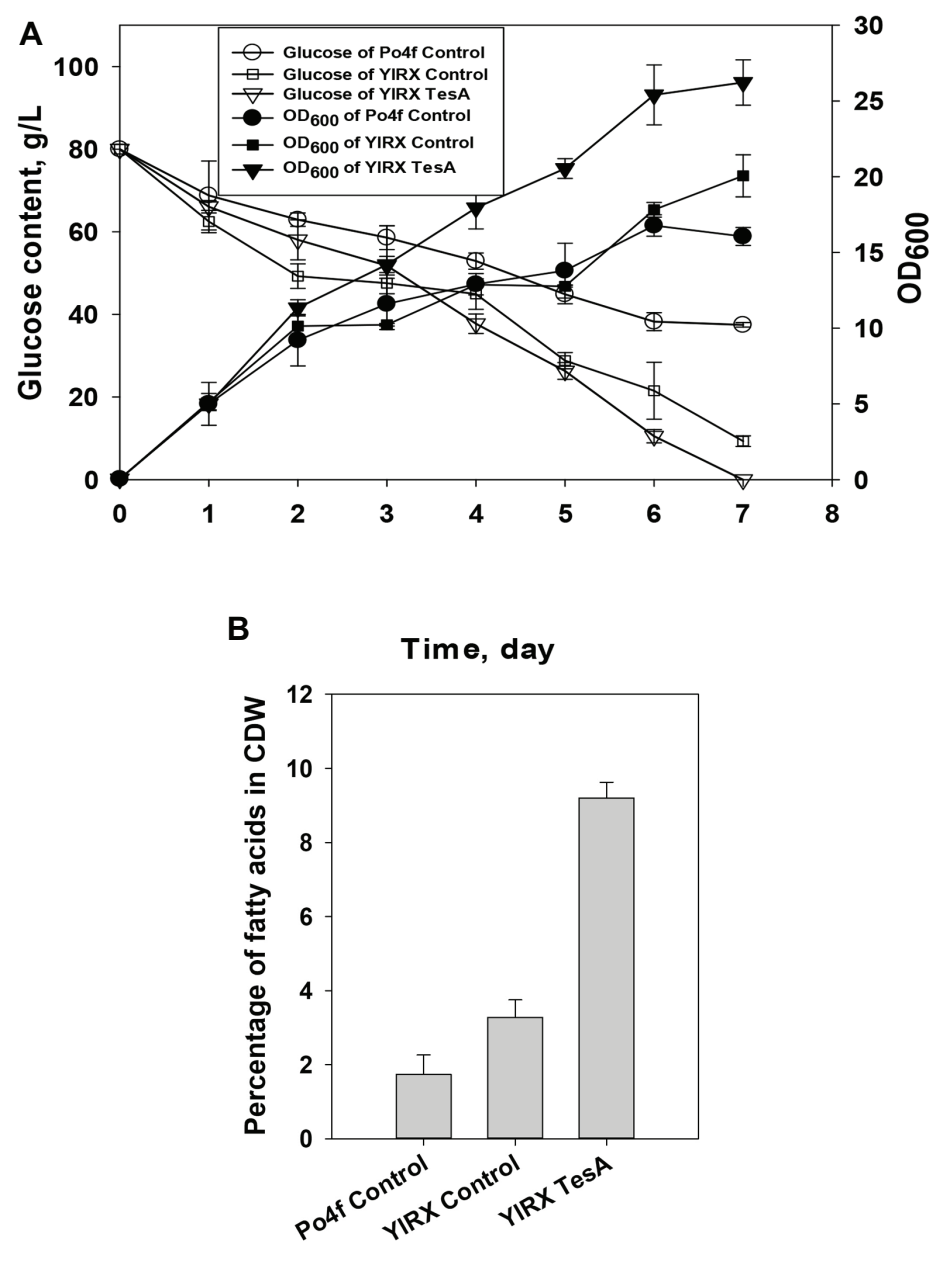
**(A)** Time function of cell growth and glucose consumption of *Y. lipolytica* strains including Po4f Control, YlRX Control, and YlRX TesA, and **(B)** Fatty acid content in *Y. lipolytica* Po4f Control, YlRX TesA, and YlRX Control grown for 5 days on the basis of cell dry weight (CDW) (wt/wt).

For complete characterization of fatty acid-overproducing strains, glucose consumption, and cell growth during fatty acid accumulation were measured (Figure 4A). The glucose concentration maintained at 80 g/L at the early stage of the cultivation of *Y. lipolytica* YlRX TesA. *Y. lipolytica* expressing *tesA* gene completely consumed glucose by day 7, but there was 37.45 and 9.3 g/L of residual glucose could be detected in the culture media of Po4f Control and YlRX Control, respectively. It was observed that *Y. lipolytica* YlRX TesA had higher biomass yield than the other two strains. Neither the strain YlRX Acc1 nor YlRX Hexo used up glucose at the end of culture period, and there was around 7 g/L glucose left in the culture media (Supplementary Figure S1). The reason for growth improvement after expression of TesA is not clear but may be attributed to rapid conversion of inhibitory acyl-CoA molecules such as steroyl-CoA and palmityl-CoA to their counterparts of fatty acids. The results also indicated that fatty acid content in *Y. lipolytica* YlRX TesA was much higher than the contents in strains Po4f Control and YlRX Control, up to 9.2% of CDW on day 5 (Figure 4B).

**Figure 5:**
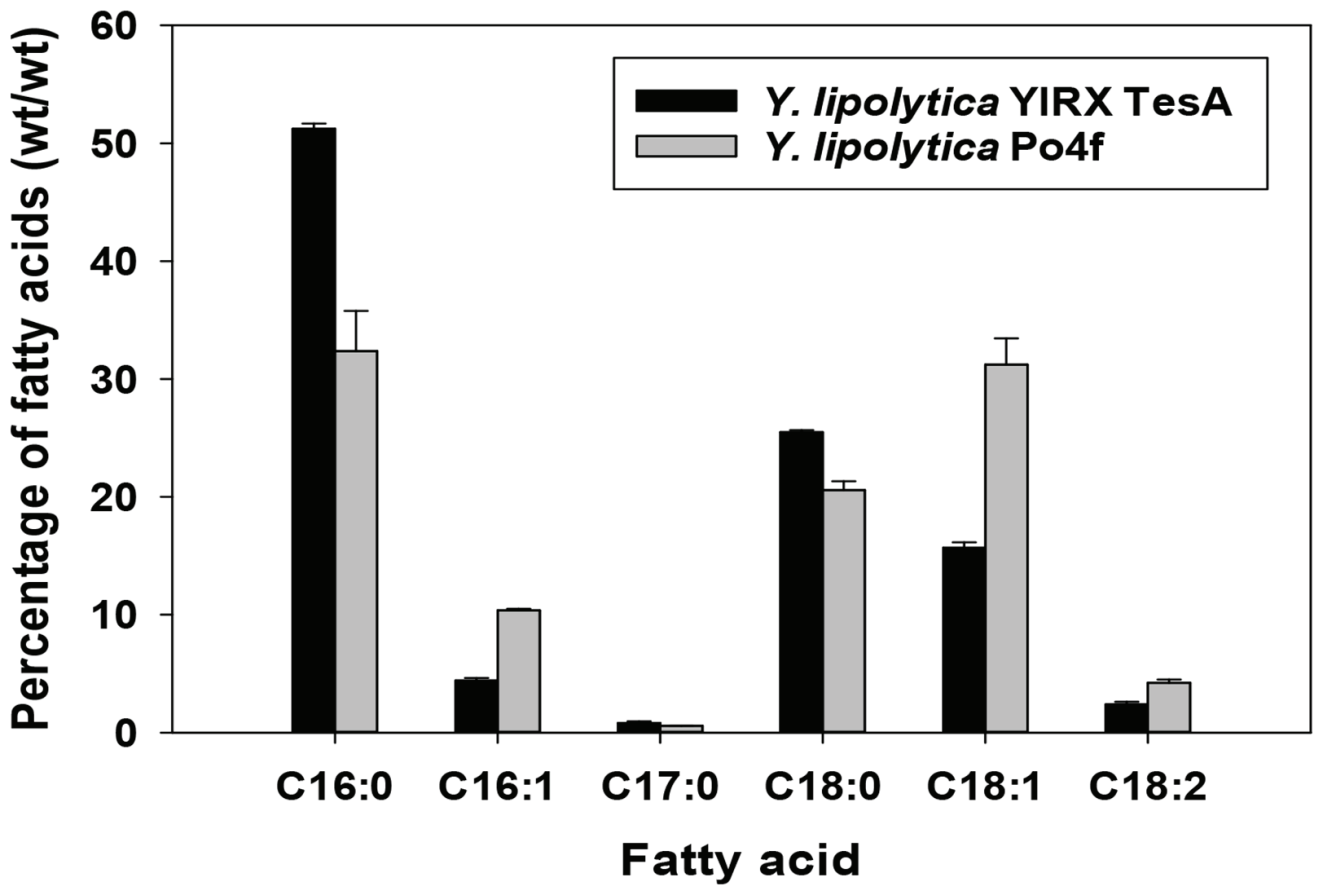
Profile of fatty acid in *Y. lipolytica* YlRX TesA and control strain Po4f cultured for 5 days. The fatty acid composition profile was shown as percent (wt/wt). The carbon source is indicated in parentheses after the strain name. C16:0, palmitic acid; C16:1, palmitoleic acid; C17:0, heptadecylic acid; C18:0, stearic acid; C18:1, oleic acid; and C18:2, linoleic acids. Fatty acids were quantified using external standards with gas chromatography (GC) analysis.

An obvious change in the composition of fatty acids was observed in the recombinant *Y. lipolytica* YlRX TesA (Figure 5). There was a remarkable increase in saturated fatty acids, including palmitic acid (C16:0) and stearic acid (C18:0), in strain YlRX TesA compared with those in the control strain Po4f. The amount of saturated fatty acids was ~3.5 times higher than that of unsaturated fatty acids. Heptadecylic acid (C17:0), a very rare metabolite in the cells, was detected. In the strain engineered for overproduction of lipids, production of heptadecylic acid was also observed, perhaps, due to biosynthesis of less favored odd-chained fatty acid resulted from active lipogenesis (Blazeck et al., 2014). In addition, high lipid titers also allowed measurement and detection of fatty acids with low abundance.

**Figure 6:**
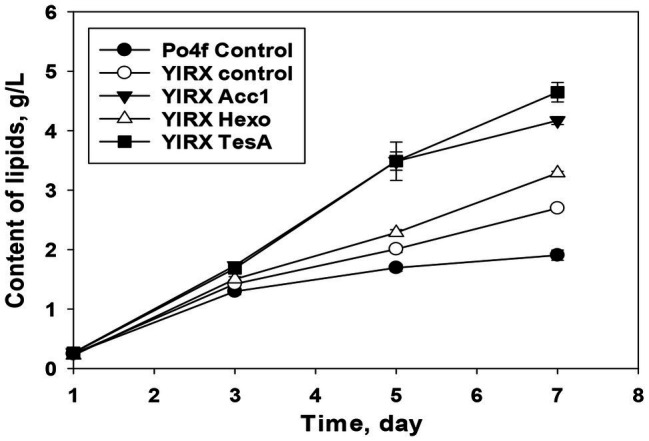
Time function of lipid accumulation in the developed recombinant strains overexpressing *YlAcc1*, *YlHxk1*, and *tesA* from *E. coli*. *Y. lipolytica* Po4f Control and YlRX Control were also cultured under the same conditions. Lipids consisted of free fatty acids, TAGs, steryl esters, and other intermediates containing fatty acid group.

Regarding the accumulation of lipids in the recombinants developed in this study, *Y. lipolytica* YlRX TesA reached the highest lipid content of 4.64 g/L on day 7 (Figure 6). Lipid concentration increased in all the modified strains compared with the parent strain. The significant decrease of fatty acid content but an increase in lipid content was observed on day 7 in *Y. lipolytica* YlRX TesA, suggesting rapid turnover between fatty acids and lipids. The similar pattern for interconversion of fatty acids into lipids was not observed in the strain YlRX Control (Supplementary Figure S2). Both YlRX Acc1 and YlRX Hexo accumulated more lipids than the control strain and the lipid content reached 4.17 and 3.2 g/L, respectively (Figure 6). These results demonstrated that *Y. lipolytica* had a great potential to accumulate fatty acids and other derivates.

## Discussion

Results from this study demonstrated the feasibility of metabolic engineering of oleaginous yeast, *Y. lipolytica*, to overproduce fatty acids. The strains were modified by rewiring lipid metabolism to produce long-chain fatty acids as the targeted products. The major metabolic engineering approaches included impairing fatty acid activation and degradation pathways and expression of bacterial thioesterase for hydrolysis of acyl-CoA into fatty acids. The maximum fatty acid titer of 2.3 g/L was achieved under a shake flask culture condition. The developed strain showed a substantial increase in saturated fatty acids. This property is potentially beneficial for production of biodiesel due to better oxidative stability and higher combustion value of FAMEs consisting of saturated fatty acids than unsaturated fatty acids (Rasoul-Amini et al., 2011). Although the produced fatty acids were mainly accumulated in the cells without being secreted, there was no growth defect observed. Aligned with our work for metabolic engineering of *Y. lipolytica* to produce long-chain dicarboxylic acid (Abghari et al., 2017) and fatty alcohol (Wang et al., 2016), the current study further improved *Y. lipolytica* for production of long-chain (C16–C18) fatty acids.

This study explored *Y. lipolytica*’s ability of overproduction of fatty acids. Two main strategies, including deletion of *faa* genes for blocking fatty acid consumption and deletion of genes related to assembly of TAGs and steryl esters, have been implemented prior to overexpression of genes, including *YlHxk1*, *YlAcc1*, and *TesA*. It was observed that the strategy of blocking activation and peroxisomal uptake of fatty acids worked better than the elimination of TAG and steryl ester biosynthesis in terms of improving fatty acid production. Blocking TAG and steryl ester biosynthesis in *Y. lipolytica* led to the detrimental effects on cell growth, although these pathways were presented as non-essential pathways in *S. cerevisiae* (Sandager et al., 2002; Valle-Rodríguez et al., 2014). This result was consistent with the previous findings of deletion of the genes for biosynthesis of TAGs and steryl esters in *Y. lipolytica* (Beopoulos et al., 2012; Ledesma-Amaro et al., 2016). Expression of thioesterase from *E. coli* for hydrolysis of acyl-CoA into fatty acids further improved the cell growth of the strain bearing disrupted *faa* and *pxa1* genes. These results suggested that the inhibition to the cell growth was not mainly attributed to fatty acids but acyl-CoA accumulated in the cells. Although four candidate *faa* genes from *Y. lipolytica* were identified based on their identities to the corresponding genes from *S. cerevisiae*, two of them were not fully characterized for their biochemical roles in fatty acid utilization (Dulermo et al., 2015). The function of FAA enzymes can be evaluated by comparison of growth on fatty acids with different chain lengths, lipid and fatty acid contents, and enzymatic activities of individual and combinational knockouts bearing disrupted *faa* genes. Alternatively, the substrate preference of FAA enzyme can be investigated by expression of the targeted gene in a *S. cerevisiae* mutant deficient of all the native *faa* genes and observation of the growth of recombinant bearing a specific *faa* gene from *Y. lipolytica* on fatty acids with different chain lengths (Knoll et al., 1995). It is also important to understand the relationship of the transcriptional regulation of *faa* genes between lipogeneses (Liu et al., 2019). The localization of FAA enzymes in cellular compartments such as cytosol and peroxisome can be tracked by using fluorescent protein tagging (Dulermo et al., 2016; Bredeweg et al., 2017). Importantly, compartmentalization of enzymes for biosynthesis of lipid-related compounds to the yeast organelles such as peroxisome and endoplasmic reticulum instead of cytosol was beneficial to achieve higher titers and yields for production of oleochemical including fatty acids (Xu et al., 2016; Yang et al., 2019).

As an oleaginous yeast, *Y. lipolytica* is uniquely advantageous for production of fatty acids and fatty acid-based chemicals by capitalizing on advancements in synthetic biology. The results in this study provide insights into improvement of *Y. lipolytica* for fatty acid production by metabolic engineering. The results add new information and advance the current understanding of mechanism underlying fatty acid and lipid metabolism. Although the oleaginous yeast *Y. lipolytica* can generate a high carbon flux toward cytosolic acetyl-CoA under nitrogen limiting culture conditions, increasing acetyl-CoA pool further improved oleochemical production (Xu et al., 2016). The performance of the strains developed in this study needs to be further improved. The future work will include pathway optimization and evolution of the stains for target production, optimization of the cultivation conditions including fed-batch cultivation by using bioreactor, expression of genes encoding efflux pumps for secretion of produced fatty acids to the supernatant (Doshi et al., 2013), and manipulation of regulatory factors governing fatty acid and lipid biosynthesis (Seip et al., 2013).

## Data Availability Statement

The raw data supporting the conclusions of this article will be made available by the authors, without undue reservation.

## Author Contributions

XX conceived the idea, and XX and SC supervised the work. XX and RG planned the experiments and conducted the experiments including molecular cloning and construction of yeast recombinants. RG collected and analyzed the data. All authors contributed to the article and approved the submitted version.

### Conflict of Interest

The authors declare that the research was conducted in the absence of any commercial or financial relationships that could be construed as a potential conflict of interest.
