# Supplementary material for: Metabolic Engineering of Oleaginous Yeast Yarrowia lipolytica for Overproduction of Fatty Acids
Source: Front Microbiol. 2020 Jul 24;11:1717. doi: 10.3389/fmicb.2020.01717 (PMC7418586; doi:10.3389/fmicb.2020.01717)

Supplementary Material

Metabolic Engineering of Oleaginous Yeast *Yarrowia lipolytica* for Overproduction of Fatty Acids

Rishikesh Ghogare, Shulin Chen, Xiaochao Xiong*

Department of Biological Systems Engineering, Washington State University, Pullman, WA 99164-6120, USA

***Correspondence:**

Corresponding Author: Dr. Xiaochao Xiong, E-mail: xcxiong@wsu.edu

Phone: +1 509-335-5996; Fax: +1 509-335-2722

Table S1 Primers used in this study.

Primers	Sequences (5’-3’)	Purpose
Yl FAA1 UP 1	ctattgggcccacatgataagcctcct	Generation of knockout plasmid
Yl FAA1 UP 2	gcgcatctagattatgccgtgggtt	Generation of knockout plasmid
Yl FAA1 DOWN 1	gccatggatcctctgacagttttgca	Generation of knockout plasmid
Yl FAA1 DOWN 2	gcttactgcagtcccgaagattccctat	Generation of knockout plasmid
Yl FAA1 TEST F	atcaggccgtgcacaaaga	Screening and verification of yeast knockout
Yl FAA1 TEST R	ggtgacgatagccgaaaac	Screening and verification of yeast knockout
Yl FAA3 UP 1	ggatagggcccttccgacgaacctgtttccg	Generation of knockout plasmid
Yl FAA3 UP 2	gggcatctagagacggtgcatgaacccattg	Generation of knockout plasmid
Yl FAA3 DOWN 1	gcatcactagtcccattgagcttgacggagt	Generation of knockout plasmid
Yl FAA3 DOWN 2	acgtccatatgctcggttcttcatgccctgt	Generation of knockout plasmid
Yl FAA3 TEST F	ctgagtctccagctcgctaa	Screening and verification of yeast knockout
Yl FAA3 TEST R	tgagcactttcggtgagacc	Screening and verification of yeast knockout
Yl FAA4 UP 1	agcttgggccccctgccatgtatgttgtaga	Generation of knockout plasmid
Yl FAA4 UP 2	ccggctctagattccagaagacatggccagg	Generation of knockout plasmid
Yl FAA4 DOWN 1	gcatcactagtctgagctggaagacgcgtta	Generation of knockout plasmid
Yl FAA4 DOWN 2	gcatacatatgtgctggacctgaaagctac g	Generation of knockout plasmid
Yl FAA4 TEST F	agaatacgccctttccgcgc	Screening and verification of yeast knockout
Yl FAA4 TEST R	caagtctggcggtgtcgagg	Screening and verification of yeast knockout
Yl PXA1 UP 1	gtattgggcccatatcggttccctttca	Generation of knockout plasmid
Yl PXA1 UP 2	gcgcttctagagacgtttgccatgatg	Generation of knockout plasmid
Yl PXA1 DOWN 1	cgattggatccaagaagcgaaagaccga	Generation of knockout plasmid
Yl PXA1 DOWN 2	cgcatctgcagacactcttcatgtatcc	Generation of knockout plasmid
Yl PXA1 TEST F	ttaatgcctatcagcgccg	Screening and verification of yeast knockout
Yl PXA1 TEST R	ttcggcgcttccacatatc	Screening and verification of yeast knockout
Yl FAT1 UP 1	ggatagggcccaatggaacgagtttcaatg	Generation of knockout plasmid
Yl FAT1 UP 2	gcgaatctagaggtgatagtggagaagggga	Generation of knockout plasmid

Yl FAT1 DOWN 1	gccatactagtcaagaaccagaagatgcccc	Generation of knockout plasmid
Yl FAT1 DOWN 2	gggatcatatggagagatgtagatggtcggc	Generation of knockout plasmid
Yl FAT1 TEST F	aaatcgagaacacggggaac	Screening and verification of yeast knockout
Yl FAT1 TEST R	cgccatctttgtgttcgtct	Screening and verification of yeast knockout
Yl DGA1 UP1	cgacg ggccctggtgcatttttgcttgcgat	Generation of knockout plasmid
Yl DGA1 UP2	cgaactctagatgggagcttatcagtcacgg	Generation of knockout plasmid
Yl DGA1 DOWN1	ctattactagt ggaaaactgcctgggttagg	Generation of knockout plasmid
Yl DGA1 DOWN2	caa cta gtagatgaccctgacgcagatg	Generation of knockout plasmid
Yl DGA1 TEST F	gtatcaacagcccgtcccaa	Screening and verification of yeast knockout
Yl DGA1 TEST R	accgtcaccgagcattttct	Screening and verification of yeast knockout
Yl DGA2 UP1	atgcagcatgctatcttcccacgtttgtata	Generation of knockout plasmid
Yl DGA2 UP2	gctattctagaacgactatgagcaagcctga	Generation of knockout plasmid
Yl DGA2 DOWN1	cgcttactagtaagcacgtgatccgaaacct	Generation of knockout plasmid
Yl DGA2 DOWN2	cctgtcatatgtcgccacgatctgtactcct	Generation of knockout plasmid
Yl DGA2 TEST F	tggcgtcaaatggcactaca	Screening and verification of yeast knockout
Yl DGA2 TEST R	taagccgggttattggcgtt	Screening and verification of yeast knockout
Yl LRO1 UP1	cgatcgggcccggaaccagacttgctccaca	Generation of knockout plasmid
Yl LRO1 UP2	cgatatctagaaccggggtagctgagacat	Generation of knockout plasmid
Yl LRO1 DOWN1	gcataactagt caatcccgagcagaccaact	Generation of knockout plasmid
Yl LRO1 DOWN2	acgtaactagtaccctggaatgtgaagcgag	Generation of knockout plasmid
Yl LRO1 TEST F	tgtcaactcctccagccaag	Screening and verification of yeast knockout
Yl LRO1 TEST R	atacgagcgtcctgtccttg	Screening and verification of yeast knockout
Yl ARE1 UP1	ttacggcatgcagcaatacggttcctggacg	Generation of knockout plasmid
Yl ARE1 UP2	gtacgtctagactcccgcactatcgagtgtc	Generation of knockout plasmid
Yl ARE1 DOWN1	gccatactagttctgggacgccattctcaac	Generation of knockout plasmid
Yl ARE1 DOWN2	gggatcatatggaccgccgtgctaaaaagac	Generation of knockout plasmid

Yl ARE1 TEST F	caatgcagccaacaacgtca	Screening and verification of yeast knockout
Yl ARE1 TEST R	ttgcggctaaaataggtgcc	Screening and verification of yeast knockout
Yl URA3 TEST F	tcctggaggcagaagaactt	Screening and verification of yeast knockout
Yl URA3 TEST R	agcccttctgactcacgtat	Screening and verification of yeast knockout
Yl ACC1 F	gccatcccgggcgactgcaattgaggacact	Gene expression
Yl ACC1 R	cccttcccgggtcacaaccccttgagcagct	Gene expression
Yl HXK F	gcatgcccggggttcatcttggtccc	Gene expression
Yl HXK R	gctagcccgggctaaatatcgtacttgacac	Gene expression
Ec TESA F	ccatcaagcttgcagcggacacgttatt	Gene expression
Ec TESA R	gctcgcccgggttatgagtcatgattta	Gene expression

Table S2 Comparison of identities of Faa1, Faa2, Faa3 and Fat1 from *Yarrowia lipolytica* with the corresponding genes from *Saccharomyces cerevisiae*.

	YlFaa1 (YALI0D17864g)	YlFaa2 (YALI0E12859g)	YlFaa3 (YALI0F06556g)	YlFat1 (YALI0E16016g)
*Sc*Faa1 (YOR317W)	50%			
*Sc*Faa2 (YER015W)	27%			
*Sc*Faa3 (YIL009W)	49%			
*Sc*Faa4 (YMR246W)		24%	27%	
*Sc*Fat1 (YBR041W)		23%		45%
*Sc*Fat2 (YBR222C)		52%	24%	

Figure S1. Comparison of glucose consumption between recombinant strains YlRX, YlRXAcc1 and YlRX Hexo

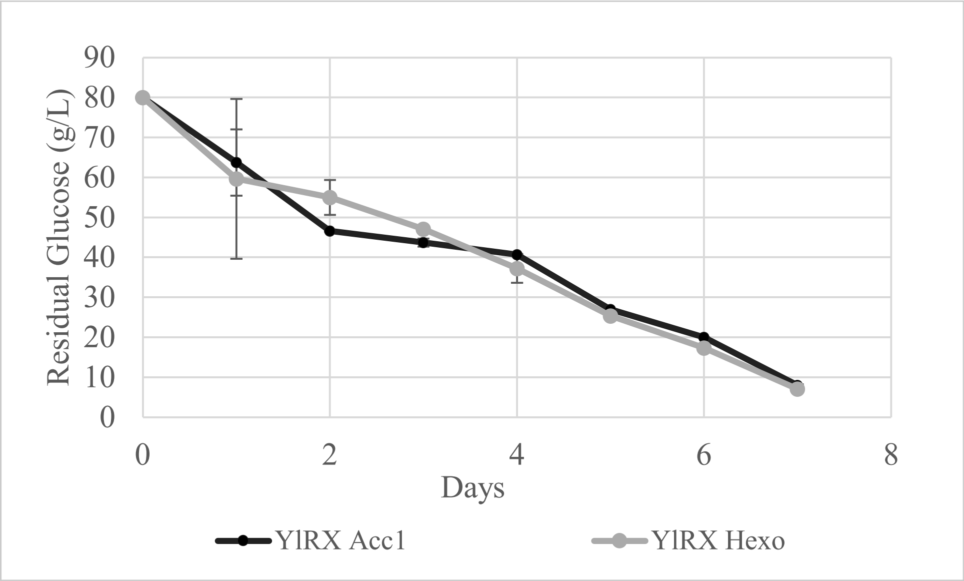

Figure S2. Ratio of free fatty acid to total lipid for Po4f control strain and YlRX TesA mutant. Results are the mean of duplicate experiments, and error bars indicate standard deviations.

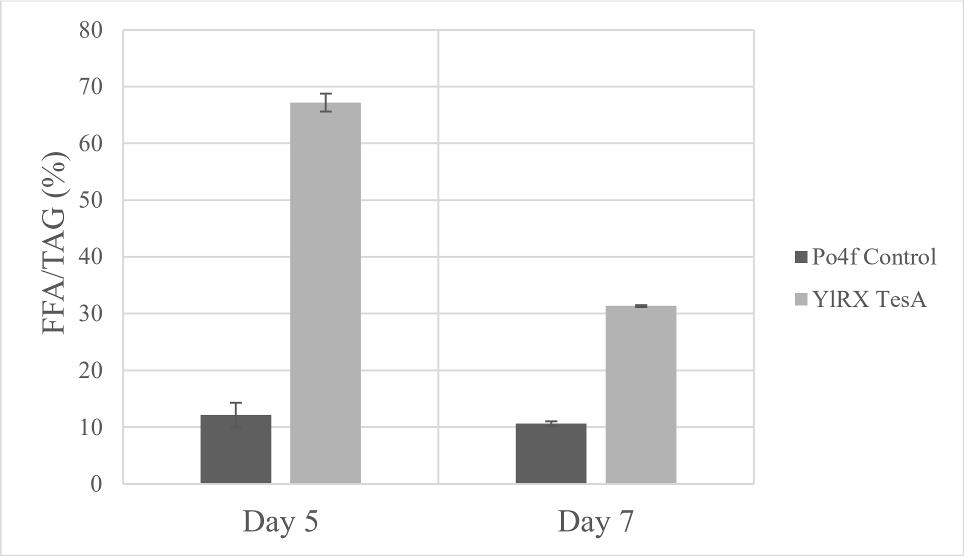

Supplement: Supplementary file 1 [file Data_Sheet_1.PDF]
